# The relationship of nursing home price and quality of life

**DOI:** 10.1186/s12913-020-05833-y

**Published:** 2020-11-09

**Authors:** Sarah Raes, Sophie Vandepitte, Delphine De Smedt, Herlinde Wynendaele, Yannai DeJonghe, Jeroen Trybou

**Affiliations:** Department of Public Health and Primary Care, Ghent University, Ghent, Belgium

**Keywords:** Quality of life, Price, Nursing homes, InterRAI, Ownership type

## Abstract

**Background:**

Knowledge about the relationship between the residents’ Quality of Life (QOL) and the nursing home price is currently lacking. Therefore, this study investigates the relationship between 11 dimensions of QOL and nursing homes price in Flemish nursing homes.

**Methods:**

The data used in this cross-sectional study were collected by the Flemish government from years 2014 to 2017 and originates from 659 Flemish nursing homes. From 2014 to 2016, data on the QOL of 21,756 residents was assessed with the InterRAI instrument. This instrument contains 11 QOL dimensions. Multiple linear regression analyses were conducted to examine the research question.

**Results:**

The multiple linear regressions indicated that a 10 euro increase in the daily nursing home price is associated with a significant decrease (*P* <  0.001) of 0.1 in 5 dimensions of QOL (access to services, comfort and environment, food and meals, respect, and safety and security). Hence, our results indicate that the association between price and QOL is very small. When conducting a subgroup analysis based on ownership type, the earlier found results remained only statistically significant for private nursing homes.

**Conclusion:**

Our findings show that nursing home price is of limited importance with respect to resident QOL. Contrary to popular belief, our study demonstrates a limited negative effect of price on QOL. Further research that includes other indicators of QOL is needed to allow policymakers and nursing home managers to improve nursing home residents’ QOL.

## Background

Clinicians, researchers and policy makers increasingly acknowledge quality of life (QOL) as an important health outcome, especially for elderly patients and nursing home residents [1, 2]. Knowledge about QOL is important, as better QOL is associated with less comorbidity [3] and less depressive symptoms [4]. In literature, QOL has many definitions. Psychology defines QOL as “a conscious cognitive judgement of satisfaction with one’s life” [5]. Meanwhile, the WHO defines QOL as “the ‘individuals’ perception of their position in life in the context of the culture and value systems in which they live and in relation to their goals, expectations, standards and concerns” [6]. Researchers apply these definitions when constructing a QOL measure. Increasingly, researchers measure QOL with a multidimensional construct focusing on the social, psychological, environmental and functional aspects of the daily lives of individuals, rather than on a one-dimensional construct [7].

Entering a nursing home is a major change for nursing home residents. Still, health care professionals pay little attention to residents’ QOL. Instead, quality of care is of much higher priority to these professionals [8]. Not only is it important that QOL receives more of their attention, health professionals and nursing home managers have also the responsibility to safeguard and, if possible, improve residents’ QOL, as QOL is linked with residents’ physical and cognitive functioning [1]. To do so, health professionals and nursing home managers need to know on which indicators they should focus in order to maintain the QOL in nursing homes as much as possible. A study focusing on this topic concluded that QOL is affected by resident characteristics (age, gender, education or chronical conditions) and showed that physical disabilities, mental health problems, and age are negatively associated with the residents’ QOL [7]. Besides resident characteristics, research has also proven that QOL is affected by facility characteristics like facility size [7, 9], ownership type [10], nursing home staffing rates [11] and financial characteristics [7]. For instance: past research indicated that ownership type might explain QOL as residents in smaller and public nursing homes report better QOL [10]. The latter might be driven by the quality of the provided care, as private nursing homes tend to provide significantly lower quality of care than public nursing homes [10, 12]. Also, the sources of payment upon admission (like the percentage of residents on Medicaid, Medicare or self-pay insurance), seem to be associated with QOL, but results on this association are inconclusive [7].

Not only is QOL associated with resident and facility characteristics, researchers also showed that it is associated with the quality of care provided by the care staff [7, 13]. In the case of dementia for instance, care staff treating elderly people with respect and dignity and being supportive to these residents tends to be positively associated with a higher QOL of these patients [13]. In addition, another study found an association between QOL and the patient satisfaction with the care they receive [14]. This might explain why an association exists between QOL and quality of care.

Furthermore, research suggests the existence of a relationship between price and quality of care. When the price increases, nursing homes can recruit more well-trained staff and buy more suited equipment which improves the service quality in nursing homes [15, 16]. In addition, price is also negatively associated with the use of psychotropic medications [16]. The authors reported that a misuse of these medications is more frequently reported in low cost nursing homes [17], which has adverse effects on morbidity and mortality in elderly [18, 19].

As positive associations exist between QOL and quality of care and between quality of care and price, higher prices might also be associated with higher QOL. However, little is known about the direct association between price and QOL. Hence, the objective of this study is to examine the direct association between price and QOL in nursing homes.

## Methods

### Data collection and variables

The data in this study are obtained using three data sources. First, price data of nursing homes in 2017 were collected by the Agency for Care and Health of the Flemish government and contained price information of 803 nursing homes in Flanders [20]. This price was based on the daily price for a single room and included some mandatory costs like the cost of living, meals and the cost of nursing care. Besides these costs, the nursing home could also charge some additional costs for the use of internet, telephone or for medicines. Additionally, the data source also contained information on the capacity (number of beds) and the ownership type of the nursing homes. The study distinguishes two types of ownership: public and private nursing homes.

Second, a variable for the dependency rate of the nursing home residents is included. This variable is calculated by dividing the number of beds for residents with high-care needs by the total capacity, using data documented by the Agency Care and Health [21]. Data on 687 nursing homes were available in this database.

Third, QOL data in nursing homes were obtained by a study of the Agency Health and Care [22]. This survey was designed to give residents in nursing homes the opportunity to share their perceptions about the provision of care and services (from 2014 until 2016) [23]. The number of participants of each nursing home were chosen based on the size of the facility. On average, 30 residents without cognitive problems (e.g.: without daily problems with time and place determination) were voluntary interviewed in each nursing home. The interviews were self-reported. Since only residents without cognitive problems took part, results cannot be generalized to all residents. The questionnaire itself has been set up using the “InterRAI Nursing Home Quality of Life” questionnaire. The InterRAI QOL instrument consists of 53 statements covering 11 domains: access to services, activities, autonomy, comfort and environment, empowerment and support, food and meals, personal relationships, privacy, respect, safety and security, and staff-resident bonding. These 11 dimensions reflect several items from Maslow’s hierarchy of human needs, such as physiological needs (food and comfort), safety (freedom from harm), love (affection and meaningful relationships), self-esteem (being appreciated by others) and self-actualization (self-fulfillment and learning) [24]. An example of a questionnaire statement per domain is presented in Table 1.

**Table 1:** Quality of life domains and questionnaire statements

Domain	Nr. of statements	Example of a questionnaire statement
Access to Services	6	I can receive the health care that I need
Activities	5	In the previous week, I took part into meaningful activities
Autonomy	7	I decide for myself what I do with my time
Comfort and Environment	6	I suffer from the noise in the nursing home
Empowerment and Support	5	I receive information about the care that I can receive here
Food and Meals	5	I have enough variety in my meals
Personal Relationships	5	It’s possible to easily make friends
Privacy	2	I can be alone when I whish
Respect	4	The staff respect the things I like and don’t like
Safety and Security	3	I feel secure when I am alone
Staff-Resident Bonding	5	Staff members take their time for a friendly talk with me

Every answer receives a score varying from 1 (“Never”) to 5 (“Always”). An average is measured for each aspect and eventually also for each domain. In total, 21,756 residents were questioned in 783 nursing homes over a period of 3 years (2014–2016). Individual data were not available, because data only exist of aggregated information per nursing home.

Combining the three databases gives us data about 803 Flemish nursing homes, of which 144 were deleted due to missing values of the variables. The final database comprises 659 nursing homes.

### Analyses

Descriptive analyses were performed and stratified by ownership type. Correlations between all regression variables were analyzed using the Pearson correlation test. To assess the association between the price and the different domains of QOL, we used multiple linear regression analyses with QOL as dependent variable, where each domain of QOL was operationalized by an ordinal variable. We estimate each domain of QOL as a function of the price, the ownership type and the dependency rate.

Some additional analyses were also conducted. First, we tested whether the interaction between price and ownership type was statistically significant by implementing the interaction variable price*ownership type into the multiple linear regression models. Second, we stratified the multiple linear regression analysis by private and public nursing homes to test whether the association between price and QOL changes depending on ownership type. These two analyses were conducted because ownership type might influence the results as study showed that QOL is affected by ownership type [10]. Third, we conducted a sensitivity analysis where we changed the daily price for a single room into the average daily price for single and multiple rooms. We performed this analysis to control whether the results change depending on which price variable we use.

The significance levels of the regression analyses are corrected with the Bonferroni correction to compare 11 regression analyses. The new significance level is 0.005. After fitting the linear regressions, the assumptions have been verified (i.e. linearity, homoscedasticity, independence, and normality) and the Variance Inflation Factor (VIF) test for multicollinearity has been conducted. All analyses were performed with R software, version 3.6.1.

## Results

### Descriptive statistics

Table 2 shows the descriptive statistics for the total sample (*N* = 659) and per ownership type. 72% of the nursing homes in the sample are private and 28% are public. In general, the Flemish nursing homes score best on privacy, respect, safety and security, access to services, and comfort and environment. The average price of a Flemish nursing home is EUR 55.59 per day (SE = EUR 6.57). To compare the means of private nursing homes and public nursing homes, we also conducted a t-test. Private nursing homes charge a significantly higher average price (average of EUR 56.32 per day) than public nursing homes (average of EUR 53.74 per day, *P* <  0.001). None of the investigated aspects of QOL were significantly different between private and public nursing homes.

**Table 2:** Descriptive statistics of the total sample and per ownership type

	Total sample (N = 659)		Per ownership type		
			Private nursing homes	Public nursing homes	
			(***N*** = 474)	(***N*** = 185)	
Variable	Mean (SE)	Range (Min-Max)	Mean (SE)	Mean (SE)	***P***
Access to Services	4.10 (0.34)	3.00–4.90	4.10 (0.34)	4.10 (0.34)	0.99
Activities	2.90 (0.49)	1.40–4.40	2.91 (0.47)	2.90 (0.52)	0.82
Autonomy	4.00 (0.36)	2.80–4.80	3.96 (0.36)	3.90 (0.35)	0.03
Comfort and Environment	4.15 (0.33)	2.30–4.80	4.13 (0.32)	4.18 (0.34)	0.10
Empowerment and Support	3.60 (0.65)	1.60–5.00	3.60 (0.65)	3.60 (0.65)	0.93
Food and Meals	3.75 (0.38)	2.60–4.80	3.75 (0.38)	3.75 (0.39)	0.94
Personal Relationships	2.59 (0.46)	1.00–4.00	2.59 (0.46)	2.60 (0.46)	0.71
Privacy	4.64 (0.30)	3.30–5.00	4.64 (0.30)	4.65 (0.29)	0.84
Respect	4.22 (0.38)	3.00–5.00	4.23 (0.38)	4.20 (0.38)	0.35
Safety and Security	4.53 (0.31)	3.40–5.00	4.53 (0.31)	4.52 (0.33)	0.78
Staff-Resident Bonding	2.90 (0.53)	1.60–4.40	2.90 (0.53)	2.89 (0.54)	0.78
Dependency rate (%)	0.62 (0.14)	0.16–1.00	0.63 (0.15)	0.61 (0.12)	0.07
Price (EUR)	55.59 (6.57)	34.57–102.35	56.32 (7.19)	53.74 (5.15)	< 0.001

The last column presents the *P*-value of the two-sample t-test

Table 3 presents the Pearson correlation matrix. Relatively high correlations can be found between the QOL dimensions. As such, the highest correlation is between the QOL domains of respect and access to services (r = 0.84, *P* < 0.001). Other high correlations can be found between personal relationships and activities (r = 0.71, *P* < 0.001), respect and comfort and environment (r = 0.70, *P* < 0.001) and security and comfort and environment (r = 0.70, *P* < 0.001). The correlations with price are relatively low, with only food and meals (r = − 0.20, *P* < 0.001), privacy (r = − 0.13, *P* < 0.001), respect (r = − 0.14, *P* < 0.001), security (r = − 0.17, *P* < 0.001), access to services (r = − 0.19, *P* < 0.001), and comfort and environment (r = − 0.18, *P* < 0.001) being statistically significant. Lastly, the correlations with ownership are all statistically insignificant.

**Table 3:** Pearson correlation coefficients for all regression variables

Variable	(1)	(2)	(3)	(4)	(5)	(6)	(7)	(8)	(9)	(10)	(11)	(12)	(13)	(14)
(1) Access to Services	1.00*	0.36*	0.34*	0.69*	0.32*	0.56*	0.33*	0.57*	0.84*	0.66*	0.32*	0.00	0.13*	−0.19*
(2) Activities		1.00*	0.31*	0.48*	0.22*	0.49*	0.71*	0.01*	0.35*	0.28*	0.55*	− 0.01	0.15*	− 0.06
(3) Autonomy			1.00*	0.48*	0.31*	0.31*	0.40*	0.38*	0.38*	0.35*	0.25*	−0.08	−0.13*	0.05
(4) Comfort and Environment				1.00*	0.49*	0.65*	0.42*	0.57*	0.70*	0.70*	0.29*	0.06	0.13*	−0.18*
(5) Empowerment and Support					1.00*	0.36*	0.26*	0.34*	0.38*	0.43*	0.29*	0.00	0.09	−0.07
(6) Food and Meals						1.00*	0.44*	0.33*	0.55*	0.56*	0.41*	0.00	0.20*	−0.20*
(7) Personal Relationships							1.00*	−0.02*	0.35*	0.23*	0.60*	0.01	−0.01	0.01
(8) Privacy								1.00*	0.55*	0.63*	−0.04*	0.01	0.05	−0.13*
(9) Respect									1.00*	0.68*	0.37*	−0.04	0.14*	− 0.14*
(10) Security										1.00*	0.23*	−0.01	0.17*	− 0.17*
(11) Staff-Resident Bonding											1.00*	0.01	0.15*	−0.03
(12) Ownership type												1.00*	−0.05	−0.18*
(13) Dependency rate													1.00*	−0.35*
(14) Price														1.00*

The numbers in the column titles refer to the variables with the same number as in the first row

* *P* < 0.005

### Regression analyses

Table 4 represents the estimates of the regression coefficients. In 5 of the 7 regression analyses, the estimate of price is statistically significant and negative. As such, a higher price is associated with significant lower QOL in: food and meals (coefficient = − 0.01, *P* < 0.001), respect (coefficient = − 0.01, *P* < 0.001), safety and security (coefficient = − 0.01, *P* < 0.001), access to services (coefficient = − 0.01, *P* < 0.001) and comfort and environment (coefficient = − 0.01, *P* < 0.001). However, the coefficient estimates are almost zero. The coefficient estimates of dependency rate are positive and statistically significant for activities (coefficient = 0.52, *P* < 0.001), autonomy (coefficient = − 0.35, *P* < 0.001), food and meals (coefficient = 0.48, *P* < 0.001), security and safety (coefficient = 0.34, *P* < 0.001), and staff-resident bonding (coefficient = 0.58, *P* < 0.001). No coefficient estimate of ownership type is statistically significant. We also tested whether the interaction variable price*ownership type was statistically significant, but this was not the case. However, we did split the regression analyses into private and public nursing homes to check whether the association between price and QOL changes depending on ownership type. The results are presented in Table 5. On the one hand, Table 5 shows that for private nursing homes, the regression estimate of price is statistically significant and negatively associated with access to services (coefficient = − 0.01, *P* < 0.001), comfort and environment (coefficient = − 0.01, *P* < 0.001), food and meals (coefficient = − 0.01, *P* < 0.001), respect (coefficient = − 0.01, *P* < 0.001) and safety and security (coefficient = − 0.01, *P* < 0.001). On the other hand, the results in Table 5 suggest that price is negatively associated with food and meals in public nursing homes (coefficient = − 0.02, *P* < 0.001).

**Table 4:** Multiple Regression analyses for all quality of life domains (N = 659)

	Access to Services		Activities		Autonomy		Comfort and Environment		Empowerment and Support		Food and Meals		Personal Relationships		Privacy		Respect		Safety and Security		Staff-Resident Bonding	
Variables	β	SE	β	SE	β	SE	β	SE	β	SE	β	SE	β	SE	β	SE	β	SE	β	SE	β	SE
Intercept	4.50*	0.15	2.61*	0.22	4.23*	0.17	4.41*	0.15	3.60*	0.30	3.91*	0.17	2.55*	0.21	4.95*	0.14	4.42*	0.17	4.63*	0.13	2.41*	0.25
Ownership type (dummy)	−0.02	0.03	0.00	0.04	− 0.07	0.03	0.03	0.03	0.00	0.00	−0.01	0.03	0.02	0.02	−0.01	0.03	−0.04	0.03	−0.02	0.03	0.00	0.04
Dependency rate (%)	0.16	0.10	0.52*	0.14	−0.35*	0.10	0.19	0.09	0.36	0.36	0.48*	0.11	−0.02	0.13	0.02	0.09	0.27	0.11	0.34*	0.09	0.58*	0.15
Price (EUR)	−0.01*	0.00	0.00	0.00	0.00	0.00	−0.01*	0.00	0.00	0.00	−0.01*	0.00	0.00	0.00	0.00	0.00	−0.01*	0.00	−0.01*	0.00	0.00	0.00
Adjusted R^2^	0.04		0.02		0.02		0.04		0.01		0.06		0.00		0.01		0.03		0.05		0.02	
F-statistic	9.31*		5.37*		5.55*		9.07*		2.27		16.07*		0.10		3.53		7.51*		12.17*		4.98*	

* *P* < 0.005

We also performed a sensitivity analysis where we changed the daily price for a single room into the average daily price for single and multiple rooms. This had no significant effect on the estimates of the regression coefficients.

Previous to the analysis, we also tested for multicollinearity using the Variance Inflation Factor (VIF) test. The VIF scores of price (VIF = 1.19), ownership type (VIF = 1.05) and dependency rate (VIF = 1.16) are very small. Therefore, we can assume that multicollinearity is not a problem. We conducted some goodness-of-fit tests for our models and present the F-statistic and adjusted R^2^ for each model (see Tables 4 and 5). Overall, the adjusted R^2^ values are very small in every regression analysis, meaning that they do not explain much of the variance of the QOL domain.

**Table 5:** Multiple Regression analyses for private and public nursing homes

	Access to Services		Activities		Autonomy		Comfort and Environment		Empowerment and Support		Food and Meals		Personal Relationships		Privacy		Respect		Safety and Security		Staff-Resident Bonding	
Variables	β	SE	β	SE	β	SE	β	SE	β	SE	β	SE	β	SE	β	SE	β	SE	β	SE	β	SE
*Private nursing homes (N = 474)*																						
Intercept	4.58*	0.18	2.63*	0.25	4.24*	0.19	4.41*	0.17	3.98*	0.35	3.87*	0.20	2.60*	0.25	4.94*	0.16	4.50*	0.20	4.66*	0.15	2.76*	0.29
Dependency rate (%)	0.08	0.11	0.41	0.16	−0.40*	0.12	0.14	0.10	0.06	0.22	0.41	0.12	−0.14	0.16	0.02	0.10	0.19	0.12	0.30	0.10	0.36	0.17
Price (EUR)	−0.01*	0.00	0.00	0.00	0.00	0.00	−0.01*	0.00	−0.01	0.00	−0.01*	0.00	0.00	0.00	−0.01	0.00	−0.01*	0.00	−0.01*	0.00	0.00	0.00
Adjusted R^2^	0.04		0.01		0.02		0.04		0.00		0.06		0.00		0.01		0.03		0.05		0.01	
F-statistic	11.54*		4.13		6.53*		8.36*		1.78		15.09*		0.75		4.22		7.62*		14.46*		2.86	
*Public nursing homes (N = 185)*																						
Intercept	4.29*	0.30	2.73*	0.46	4.22*	0.32	4.53*	0.30	2.72*	0.57	4.15*	0.34	2.61*	0.41	5.00*	0.26	4.22*	0.34	4.59*	0.29	1.56*	0.47
Dependency rate (%)	0.40	0.20	0.92*	0.31	−0.13	0.21	0.39	0.00	1.27*	0.38	0.76*	0.22	0.41	0.28	−0.06	0.17	0.54	0.23	0.46	0.19	1.22	0.31
Price (EUR)	0.01	0.00	−0.01	0.01	0.00	0.01	−0.01	0.20	0.00	0.01	−0.02*	0.01	0.00	0.01	−0.01	−0.01	− 0.01	0.01	− 0.01	0.00	0.01	0.00
Adjusted R^2^	0.03		0.05		−0.01		0.04		0.05		0.10		0.01		0.00		0.03		0.03		0.07	
F-statistic	3.86		5.36		0.51		5.03		5.62		11.58*		1.54		1.05		4.16		4.25		8.1*	

* *P* < 0.005

## Discussion

This study investigated the direct relationship between nursing home price and QOL of nursing home residents, as knowledge about this topic is currently lacking. Literature suggested that indicators such as resident characteristics (e.g.: age, education, chronical conditions) and facility characteristics (e.g.: facility size, nursing home staffing rates) are significantly associated with QOL [7, 9, 11]. The study of Shippee et al. [7] is one of the first studies that subdivides QOL in different domains, in order to have a more nuanced understanding of how indicators are associated with QOL. Therefore, they use a survey consisting of 52 items measuring 6 QOL domains (environment, personal attention, food, personal engagement, negative mood and positive mood). In this study, we chose the internationally validated and widely accepted InterRAI instrument as QOL measure because of two reasons. First, the InterRAI instrument supports multiple clinical and management applications for different audiences (ex.: care planning, quality measurement, patient safety assessment) [25]. Second, it has been developed in an international context and has shown to be reliable and valid in multiple languages (also in Dutch) and countries [25, 26].

The descriptive results suggested that average price levels are statistically different between public and private nursing homes. Therefore, ownership type was used as a control variable in the regression analyses. The multiple regression analyses suggested that a 10 euro increase in prices is associated with a significant decrease of 0.1 in QOL in access to services, comfort and environment, food and meals, respect, and safety and security. When splitting up the regression analyses in private and public nursing homes, results indicate a weak association between price and the QOL domains of access to services, comfort and environment, food and meals, respect, and safety and security, but only in private nursing homes. Nevertheless, based on this data we do not find evidence of an association between price and the varying domains of QOL. This result suggests that a price increase does not always lead to higher QOL, even though it is the popular belief. This result adds evidence to the study of Shippee et al. [7] who only investigated the association between QOL and some financial indicators. They suggest that nursing homes with a higher percentage of Medicaid-only residents (US health insurance program only for people with a limited income); compared to Medicare (US health insurance program for people older than 65 years old and younger for people with disabilities), self-pay, and privately insured residents; are associated with lower QOL in the domains of personal attention and engagement. Besides the research question, our findings also suggest that nursing homes that have more highly dependent residents are associated with higher QOL in food and meals, respect, safety and security, and staff-resident bonding (while holding the other variables constant). This result contradicts the results of other studies. One study finds that the dependency rate is negatively associated with QOL for patients with dementia [27]. Another study finds no statistically significant association between the two variables in nursing home residents [28]. The reason why dependency rate and QOL may be associated is because residents needing a high degree of care are mostly residents that have long-term chronic and multimorbid conditions, causing physical and/or mental disabilities that may affect their QOL [28]. However, for Flemish nursing homes there is a reason why dependency rate and QOL might be positively related. In Flanders, nursing homes have two sources of income: the daily price that residents pay, and a reimbursement of the Flemish government. The latter is based on the dependency rate of their residents [29]. Possibly, nursing homes spend this higher reimbursement in better patient care, which might translate in higher QOL of their residents. Another possible explanation is that this study does not include the QOL of residents with cognitive problems, and as they may be more dependent, their QOL is not reflected in our results. This might have positively influenced our results. Furthermore, our results suggest that there is no significant association between ownership type and QOL. Meanwhile, the study of Shippee et al. [7] indicate the opposite, more specifically that public ownership type was positively associated with the QOL dimension of environment. The different results between our study and the study of Shippee et al. [7] might be explained by the varying country-specific variations in ownership type. Another variable that might influence QOL, through the dependency rate, is the socioeconomic status of the residents. It is possible that residents with a higher socioeconomic status substitute nursing home care for home care. These residents might therefore wait longer, until they are more frail and care dependent, to enter a nursing home. To test the latter, we included the average income level per inhabitant of each municipality where the nursing home is situated [30]. We then calculated the correlation between the average income level and the dependency rate, but this was statistically insignificant (r = 0.01, *P* = 0.86). Thus, we found no correlation between nursing homes in areas with higher socioeconomic status and more highly dependent residents.

The findings of this study should be interpreted carefully. First of all, the means of the QOL domains in the descriptive statistics are the means of ordinal variables. It only indicates if residents are on average more or less satisfied on a QOL domain or not. The number itself does not have a meaning. Second, because of the cross-sectional design of this study we could only assess associations but no causal relationships. Third, all the nursing homes are situated in Flanders. National data on the QOL domains were not available. Therefore, the results can only be generalized on a Flemish level since the sample includes 82% of all Flemish nursing homes. Fourth, the InterRAI survey was only taken from people without cognitive problems. Due to a low response level per nursing home, residents with cognitive problems were not included in the study. Therefore, the findings cannot be generalized for residents with cognitive problems. Lastly, the sample consists of aggregated information of residents in nursing homes, but not of individual data per resident. Therefore, we lose a lot of information that could make the data more sensitive and that could uncover more relationships. Additionally, we could not control for individual differences in QOL in the same nursing home such as: health status, socio-demographic characteristics, educational level, whether residents have Alzheimer’s disease or chronic conditions, which are predictive for a number of QOL domains [31]. However, we tried to compensate this loss by including the dependency rate. Still, a considerable part of the variation in the QOL dimensions cannot be explained simply with price, ownership type and dependency rate.

The result of this study does not correspond with our hypothesis that higher price levels are associated with higher quality of care and therefore also with higher QOL. There might be several reasons for this finding. Some questions or domains in the InterRAI instrument might not translate price changes into QOL changes. For instance, the statement “I like to go outside” might be more suitable than the current statement “I can go outside when I want”, as more expensive nursing homes might be situated in more beautiful surroundings. Other questions or domains might be more suited for our study to capture the relationship between price and QOL. Furthermore, other researchers already found that facility characteristics (in comparison to resident characteristics) explain only the minority of the variability of QOL: 9% in the research of Degenholtz et al. [32] and only 3% in the study of Shippee et al. [7]. Therefore, it is not a surprise that our results suggest a weak association between price and QOL. Though other researchers suggested that resident characteristics have a stronger association with QOL than facility characteristics, we strongly suggest future researchers to focus on other facility characteristics that might be associated with QOL, while controlling for resident characteristics such as Alzheimer’s disease, the number of chronic conditions, etc. The reason is that implementing policy recommendations based on resident characteristics such as age or marital status is unrealistic, as these characteristics are non-modifiable through policy [7]. Instead, focusing on facility characteristics such as perceived quality of care or work environment characteristics (e.g.: team climate, multidisciplinary collaboration) might be more easily modifiable through policy. The study of Backhaus et al. [33] suggests that previous variables are associated with quality of care. Therefore, they might also be associated with QOL. Other variables that may influence QOL are demand and supply variables. Demand and supply variables may influence quality of care in nursing homes. For instance, research showed that competition in the nursing home market corresponds to a decrease in quality of care and a decrease in nursing home price [34]. As quality of care and QOL are related [13], competition and other variables related to quality of care might also influence QOL. Investigating these and other variables may help to improve the QOL of nursing home residents and enable managers and policy makers to select better targeted improvement strategies.

## Conclusion

To our knowledge, this is the first study to investigate the relationship between nursing home price and residents’ QOL. Our results suggest only a significantly small and negative association between price and the QOL domains of access to services, comfort and environment, food and meals, respect, and safety and security. Although the associations are statistically significant, they are very small. Therefore, we do not find evidence of a relationship between price and QOL. In other words, our findings suggest that higher nursing home prices do not always translate into higher QOL of its residents. Further research that includes other indicators of QOL is needed to allow policymakers and nursing home managers to improve nursing home residents’ QOL. Additionally, we believe that future QOL research should further explore associations with facility characteristics rather than with resident characteristics, as facility characteristics might be more easily modifiable through policy than resident characteristics.

## Data Availability

The datasets supporting the conclusions of this article are publicly available on the website of the Flemish government, (https://www.zorg-en-gezondheid.be/publicaties) and (https://www.zorg-en-gezondheid.be/cijfers).

## Abbreviation

QOL: Quality of Life
